# Gestational Sympathetic Stress Programs the Fertility of Offspring: A Rat Multi-Generation Study

**DOI:** 10.3390/ijerph19053044

**Published:** 2022-03-05

**Authors:** Beatriz Piquer, Freddy Ruz, Rafael Barra, Hernan E. Lara

**Affiliations:** 1 Centre for Neurobiochemical Studies in Neuroendocrine Diseases, Laboratory of Neurobiochemistry, Department of Biochemistry and Molecular Biology, Faculty of Chemistry and Pharmaceutical Sciences, Universidad de Chile, Independencia, Santiago 8380492, Chile; bpiquer@ciq.uchile.cl (B.P.); freddy.ruz@ciq.uchile.cl (F.R.); rafael.barra@usach.cl (R.B.); 2 Centro de Investigación Biomédica y Aplicada (CIBAP), Escuela de Medicina, Facultad de Ciencias Médicas, Universidad de Santiago de Chile, Santiago 9170020, Chile

**Keywords:** programming, ovary fertility, sympathetic stress

## Abstract

The exposure to sympathetic stress during the entire period of gestation (4 °C/3 h/day) strongly affects the postnatal reproductive performance of the first generation of female offspring and their fertility capacity. The aim of this work was to determine whether this exposure to sympathetic stress affects the reproductive capacity of the next three generations of female offspring as adults. Adult female Sprague–Dawley rats were mated with males of proven fertility. We studied the reproductive capacity of the second, third, and fourth generations of female offspring (the percentage of pregnancy and the number and weight of female offspring). The estrus cycle activity of the progenies was studied, and a morphological analysis of the ovaries was carried out to study the follicular population. The second generation had a lower number of pups per litter and a 20% decrease in fertile capacity. The estrus cycle activity of the third generation decreased even more, and they had a 50% decrease in their fertile capacity, and their ovaries presented polycystic morphology. The fourth generation however, recovered their reproductive capacity but not the amount of newborns pups. Most probably, the chronic intrauterine exposure to the sympathetic stress programs the female gonads to be stressed in a stressful environment; since the fourth generation was the first born with no direct exposure to stress during development, it opens studies on intrauterine factors affecting early follicular development.

## 1. Introduction

All living organisms are moldable in their first moments of life and during pregnancy, so they adapt to the conditions they find in the placental environment. This flexibility allows them to adapt to the surrounding environment without being influenced exclusively by their genetic load, which is much faster than genetic adaptation [1,2,3]. Therefore, if chronic stress occurs during the gestation period, this stress is capable of modifying the placental environment and producing lasting changes in the embryos, which may affect the development of organs, the physiology, and the metabolism of the organism, something known as fetal programming [4,5].

Stress is defined as any stimulus capable of altering the organism’s homeostasis; it is necessary for survival and is the organism’s way of dealing with a threatening situation. Stress can activate various pathways: the hypothalamic–pituitary–adrenal (HPA) axis, which produces the release of adrenocorticotropic hormone (ACTH) and adrenaline (A), and/or the activation of the sympathetic nervous system (SNS), which produces the release of noradrenaline (NE). The activation of the SNS is observed in “fight or flight” situations, as would be in the case of a cold situation without hypothermia [1,6,7].

Our laboratory works with an intermittent chronic cold stress protocol (4 °C/3 h/day) applied throughout the rat gestation period or to adult rats. This stress model produces a selective sympathetic response, which allows us to know the effects of sympathetic nervous overactivation on adult rats or on the offspring of stressed rats without producing changes in the levels of A or ACTH [6,7,8]. 

We, along with other researchers [9,10,11], have described that the ovaries of patients with polycystic ovarian syndrome (PCOS, the most frequent disease in women during reproductive years), presenting with a hypernoradrenergic state characterized by an increased number of sympathetic fibers, an increased concentration of NE in the ovary, and increased sympathetic outflow. The same observations are found in rat models of the disease [12]. In addition, the increased plasma NE during pregnancy could affect the placental NE transporter (NET), which enables the maintenance of low concentrations of NE in the fetal circulation [13]. Thus, a failure in the transport produces a reduction in NE clearance, which could be the cause of an increased fetal exposure to NE. We have also described the same condition in the human placental NET in normal and in PCO gestating mothers with polycystic ovarian syndrome (PCO), the most frequent ovarian pathology in women during reproductive years [9].

In addition, the exposure to cold stress (through the activation of the sympathetic nerves) to adult rats modified the reproductive function of the female progeny when they reached adult age and induced a polycystic ovarian phenotype (the presence of ovarian cysts, hyperandrogenic condition, and decrease in corpora lutea) [14]. It also produced insulin resistance in the ovary [15]. Moreover, when it was applied to gestating female rats during pregnancy, it modified the reproductive function of the female progeny, modified early follicular development (finding a delay in the vaginal opening and less recruitment of follicles), and resulted in fewer estrus cycles [16]. 

Based on these considerations and the impact of stress as a programming factor, we propose as a working hypothesis that the in utero exposure to chronic sympathetic stress modifies the reproductive function of the progeny that are transmitted to the next generation. Thus, the main aim of the present study is to study the effect of stress exposure during gestation and the impact of this exposure in the reproductive capacity of female rats during the following three generations. We analyzed the reproductive capacity of the second, third, and fourth generations of female offspring (the percentage of pregnancy and the number and weight of females offspring). The estrus cycle activity of the progenies was studied, and a morphological analysis of the ovaries was carried out to study the follicular dynamic and the distribution of the follicular population.

## 2. Materials and Methods

### 2.1. Animals and Experimental Design

**Figure 1:**
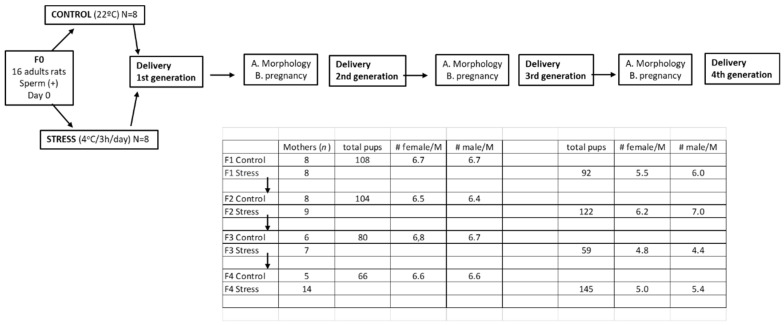
Schematic distribution of the experimental groups. The table presents the number of rats used in each study, and the scheme shows that the selection of rats of each of the daughters maintaining the same line in order to have only one rat of each of the mothers to form the group.

Female Sprague–Dawley rats weighing 250–300 g, kept at 20 °C, and with a 12:12 light–dark cycle were used as the initial rats to obtain the different generations. Food and water were constantly and freely available. The rats of the different generations were divided into three groups: One group were females selected to study the estrus cycle activity of the rats and to investigate their capacity for fertilization after mating with a male of proven fertility. The other group of females was euthanized at 60 days of age (adult stage) to fix the ovaries for morphological analysis. The remaining rats (all the males and some females) were used for other studies. Males were principally used for molecular studies in the cardiovascular system to study the impact of gestational stress to the adrenergic response in vivo. The same study in females is in progress with the females remaining from the different generations. The rats were euthanized by decapitation, and the ovaries and plasma were collected. Decapitation was performed according to the AVMA Guidelines for the Euthanasia of Animals (2020 Edition) [17]. It was performed by specialized personnel and approved by the Bioethics Committee of the Faculty of Chemistry and Pharmaceutical Sciences at the University of Chile (protocol number: CBE2016-13 to BP and CBE2017-05 to HL). The euthanasia also complied with the National Institutes of Health Guide for the Care and Use of Laboratory Animals (NIH Publications No. 8023, revised 1978) and with national guidelines (CONICYT Guide for the Care and Use of Laboratory Animals). Figure 1 graphically shows the different experimental protocols used in the biological assays.

### 2.2. Gestational Stress Induction

The chronic cold stress protocol for pregnant rats was the same as we previously used for adult rats in which we studied the first generation [13,16]. The mating procedure was as follows: Adults female rats were checked for estrus cycle activity to determine the proestrus day. On the night of the proestrus phase (when ovulation occurs), the rats were mated with males of proven fertility. The following morning, the rats were checked for the presence of a vaginal sperm plug. Rats with a vaginal plug were assigned as Day 0 of pregnancy and formed the F0 generation. The pregnancy was confirmed because after copulation, pregnant rats stop cycling, and they stay in a permanent diestrus stage. The first group of rats (F0) comprised 16 pregnant rats divided into two groups of eight control rats and eight stressed rats. The pregnant female rats from the experimental group were exposed daily to a temperature of 4 °C for 3 h a day throughout the gestation period. The control group was kept at a constant temperature of 20 °C throughout the gestation period. Dorfman et al. [14] firstly described the procedure in detail. 

### 2.3. Studies with Generations

Once the siblings were selected from each generation, they were not used in other parts of the same experiment to decrease the founder’s effect. Each of the rats used to form the minimal number of rats came from an independent F0 dam. The only exception was in F4, in which, due to the possibility that fertility would decrease even more that in F3, we increased the number to 14, including one extra rat coming from the same F0 generation.

Once the rats were born, their sex was determined, and they were assigned to foster mothers until they reached 20 days old. The total number of pups born in each progeny is shown in Figure 1. After birth, the progenies were organized having each group 12 rats each, of which half of the pups were male and the other half female (to maintain the average number of pups naturally born and to be sure that they would obtain sufficient nursing). When the rats reached 20 days old, the males were moved to use in other experiments, and the females were moved to another cages (3 per cages). In order to maintain the independence of litters, to eliminate founders effects that may impact the validity of the downstream statistical analyses, we followed each of the pregnant rats to obtain one of the rats of each mother and followed up to the next generation. We continued with the grand-daughters of the same mother for the next generation up to the fourth generation. In the Figure 1, we show the rats obtained in each generation and the average of rats in each group of rats. The rats duly marked were divided in two groups: One group was euthanized once they reached the adult age of 60 days old in order to obtain their ovaries, fix them in Bouin’s fixative, and use them for a morphology study of the ovary to identify the follicular structures of the ovary. The other group of rats were used to study their estrus cycle activity and for fertilization studies. These rats were mated on the proestrus day with males of proven fertility as described above. The rats that were not sperm positive during the proestrus day were continuously exposed to the male during the following proestrus days up to one month. After the end of the procedure, the number of pregnant rats and the number of offspring were quantified. The number of live pups was counted, and they were weighed [13,18]. As described above, the remaining rats of each generation (mainly males) were used for other experiments that had been previously designed.

### 2.4. Estrous Cycling Activities of the Rats

During the study period, the groups of rats were examined by daily vaginal smears for estrus cycle regularity. Results are presented as the number of estrus cycles with the following stages: proestrus (P), estrus (E), and metestrus–diestrus (M-D). Control rats presented regular 4–5-day estrus cycles [19,20]. A retrospective evaluation of the data from our animals showed that all control rats from our colony presented nearly 80% of a regular estrus cycle (short cycles are considered 4 days, and long cycles are considered 5 days) [21,22].

### 2.5. Morphometric Analysis

The ovaries were fixed in Bouin’s fluid, embedded in paraffin, cut into 6 µm sections, and stained with hematoxylin and eosin. Morphometric analyses of whole ovaries were performed according to the method described by Hirshfield [23] with modifications [24], using *n* = 5 ovaries for each of the experimental groups. We used the following classification: Antral follicles were those with more than three healthy GC layers, the antrum, and a clearly visible nucleus of the oocyte. Atretic follicles had more than 5% of the cells with pyknotic nuclei in the largest cross-section and exhibited shrinkage and the occasional breakdown of the germinal vesicle. Type III follicles represent pre-cystic follicular structures and were also defined according to the criteria proposed by Brawer and colleagues [25]. These follicles are large; devoid of oocytes; contain four or five plicate layers of small, densely packed granulosa cells surrounding a very large antrum; and display a seemingly normal thecal compartment. Cystic follicles were devoid of oocytes and displayed a large antral cavity, a well-defined thecal cell layer, and a thin (mostly monolayer) GC compartment containing apparently healthy cells.

### 2.6. Statistical Analysis

The number of animals for all experiments was calculated as the minimum number of animals according to the variability of the experimental procedures. The minimum number of animals was calculated according to the following equation [26]:

$$n=\frac{2{\left(Z\alpha +Z\beta \right)}^{2}\times S^{2}}{d}$$

where *n* is the number of animals for each condition, *S* is the standard deviation, *d* is the difference needed to obtain statistical significance, *Zα* is the probability of type I error (significance), and *Zβ* is the probability of type II error (power). We proposed that α = 0.05, meaning the probability of finding a statistically significant difference, was 0.05; β = 0.3, indicating the probability of having a difference between the populations; the intrapopulation variation was 0.2; and d, the smallest difference in the population, was 0.11. Thus, we obtained *n* = 4.5. Therefore, to obtain a statistically significant difference of *p* < 0.05, we needed to use at least four or five animals per study group. The α, β, intra-population variation, and d values were calculated from preliminary data from our laboratory with the same facility. Comparison between two experimental groups was performed with a *t*-test with a significant threshold of *p* ≤ 0.05. The statistical significance among several groups was tested with a one-way ANOVA followed by a Newman–Keuls post hoc test or Tukey’s multiple comparisons test, according to proper experimental design. The significance threshold was set to *p* < 0.05. To analyze difference between proportions, we used chi-square test (Prism GraphPad, La Jolla, CA, USA).

## 3. Results

### 3.1. Effect of Gestational Stress on the Estrus Cycling Activity of the Rats of the Second, Third, and Fourth Generation

**Figure 2:**
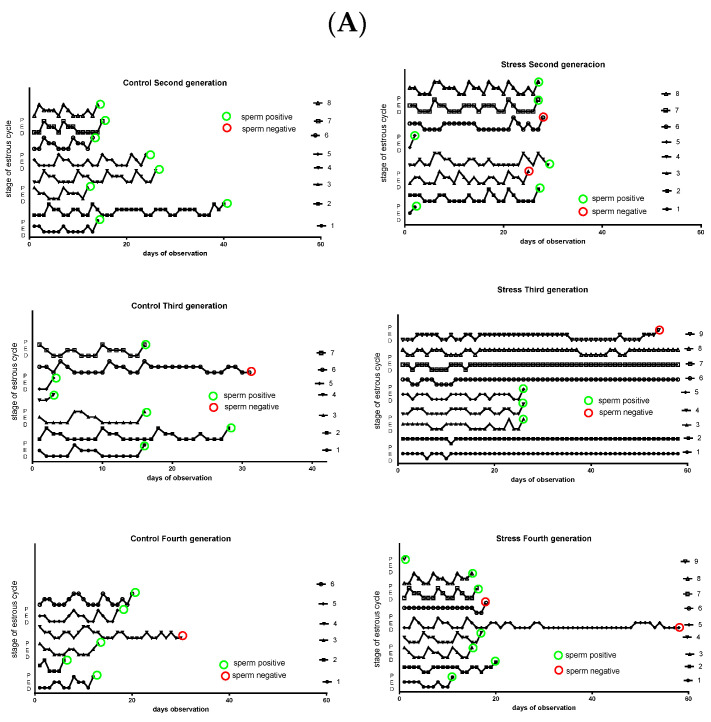

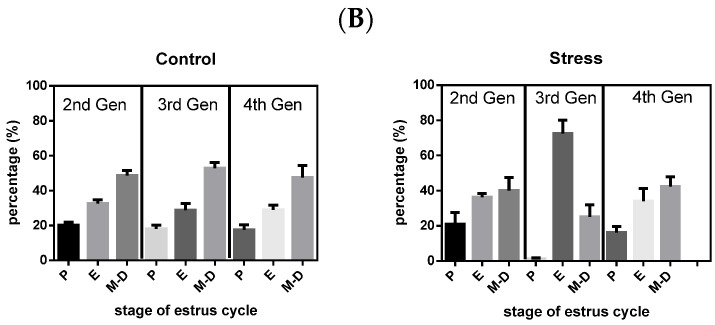
Gestational stress and its consequence on estrus cycle activity (**A**). The left side of this figure shows representative animals for each group of controls. The right side shows the cycle activity of representative animals of the stressed rats of different generations. The red circle means that during the night of proestrus, the female was mated with a male of proven fertility, but there was no copulation because there was not a sperm plug in the vagina. The green circle means that there was a sperm plug. When there is no circle, the female never accepted the male. (**B**) The percentage of the time that rats stay in each of the stage of estrus cycle along the observation period (up to observance of being sperm (+)). P, proestrus; E, estrus; M-D, metestrus–diestrus.

Figure 2 shows the estrus cycle activities of the control and stressed rats. The left side of Figure 2A shows the profile for control rats. Each rat has a characteristic cycle profile in which there regularly appeared peaks in the cycling behavior that corresponded to a proestrus stage. The green circle means that on the night of the proestrus day, the rat was mated with a male and presented with a sperm plug in the vagina (sperm positive) the next day. Almost all of the control rats were sperm positive and attained pregnancy. The red circle means that the rats were sperm positive but did not attain pregnancy. The right side shows the stressed rats across the different generations. A slight decrease in the number of sperm-positive rats was found in the second generation. This condition was clearly found in the third generation and returned to the control in the fourth generation. Because not all rats were pregnant in the first intent, rats were followed for different times up to 60 days for pregnancy. In Figure 2B are the same data but normalized as percentage of the time that rats were in the different stage of the estrus cycle. On the left side is the behavior of control rats of the second, third, and fourth generation. All the groups have a predicted behavior in that they remain in proestrus for less time (and thus in the fertile stage), followed by estrus, and almost 50% of the time were in metestrus–diestrus. However, in the stressed rats, the behavior presented many changes, especially in the third generation, in which a great percentage of the time, the rats were in estrus and spent almost no time in proestrus. The percentage of time was almost normal in the fourth generation. 

### 3.2. Effect of Gestational Stress on the Fertile Capacity

**Figure 3:**
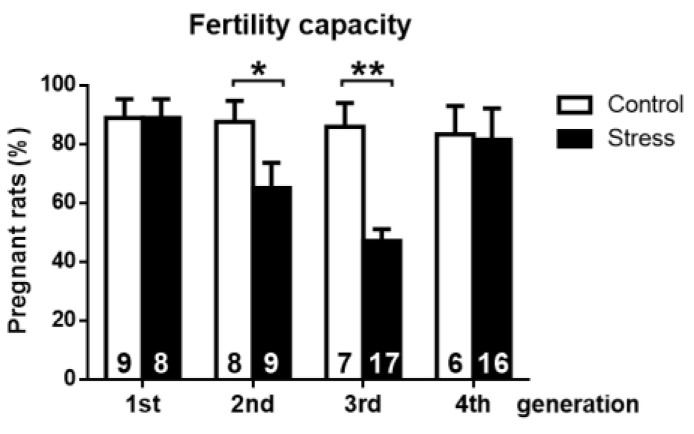
Physiological impact of gestational stress on the second, third, and fourth generation of rats. Fertility is expressed as the percentage of total rats that became pregnant after being exposed to male rats. The results are shown as the mean ± s.e.m. of the number of rats in each graph. The results were analyzed with a chi-square test. Brackets show the significance between groups. * *p*< 0.05; ** *p* < 0.01.

In Figure 3, we summarize the effect of gestational stress on the capacity of female rats of the different generations to attain pregnancy. Control rats presented the expected mating behavior, i.e., when the female was exposed to a fertile male during the night of proestrus, we found that in 80–90% of them, a sperm plug was present the next day, indicating copulation. In the stress group, we observed no changes in the percentage of pregnant rats in the first generation. In the next generation, we found a decrease in copulation because the females of the second generation presented a sperm plug in six out of eight rats. The principal effect was found when the fertile females of the third generation were mated because only 50% of the total females were sperm positive. Two out of nine of the remaining rats accepted the male, but no pregnancy appeared, and the others did not accept the male at all.

**Figure 4:**
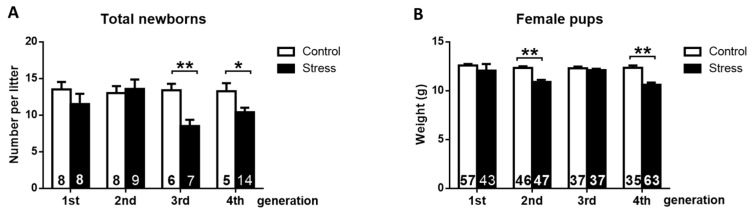
Physiological impact of gestational stress on the second, third, and fourth generations of rats. (**A**) The total number of newborn rats. In the graph, the number of pregnant rats in each group is shown. (**B**) The body weight of newborn female rats at 4 days. The results are shown as the mean ± s.e.m. of the number of rats in each graph. The results were analyzed with a one-way ANOVA followed by the Newman–Keuls post hoc test. Brackets show the significance between groups. * *p* < 0.05; ** *p* < 0.01.

Of these remaining nine rats, only three became pregnant and had live pups. Comparing the three generations of stress-exposed rats, the third generation had not only the lower rate of fertility but also the lower number of pups per animal, which was similar to the findings in the fourth generation (Figure 4).

### 3.3. Effect of Gestational Stress in Follicular Dynamics of Ovary Follicular Development 

**Figure 5:**
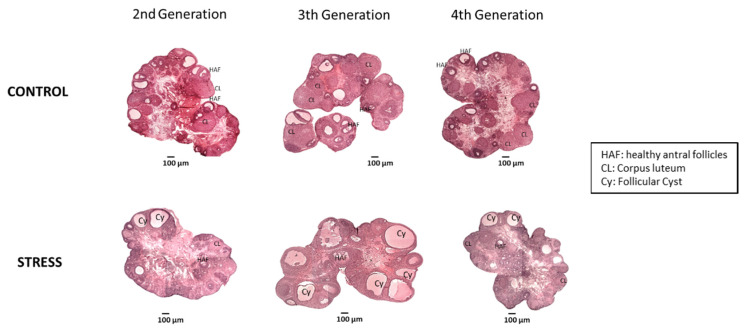
Representative picture of the effect of gestational stress on the second, third, and fourth generation of rats. The bar corresponds to 100 µm. In the images, healthy antral follicles (HAF), atretic follicles (AF), corpora lutea (CL), and cysts (C) are marked.

Cold stress during gestation produced long-term changes in follicular development and increased ovarian cysts. Figure 5 shows a representative image of central slices of the rat ovaries of the experimental groups. Twenty-eight days of chronic cold stress exposure during gestation resulted in trans-generational changes that were clearer in the third generation, characterized by an increased number of ovarian cysts and a decreased number of corpora lutea as compared with their age and generation-matched control ovaries (Figure 5). The fourth generation, however, was characterized by a reversion of the morphological changes to a control phenotype.

**Figure 6:**
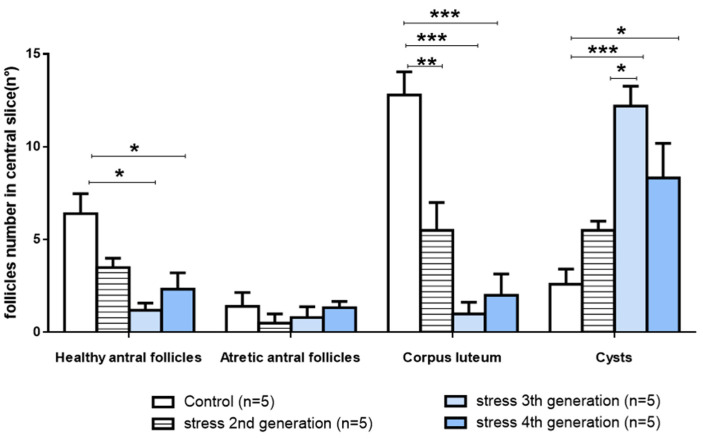
Ovarian follicular dynamic for the effect of gestational stress on the second, third, and fourth generations of rats. The number of healthy antral follicles, atretic follicles, corpus luteum, and cysts is presented. (*n* = 5); mean ± s.e.m. One-way ANOVA with Tukey’s multiple comparisons test (differences between brackets: * *p* < 0.05; ** *p* < 0.01; *** *p* < 0.001).

The quantitative analysis confirmed the initial finding of the appearance of the ovaries. There was a statistically significant decrease in the amount of healthy antral follicles and no changes in atretic antral follicles (Figure 6). The progressive decrease in the healthy antral follicles was also followed by a more pronounced decrease in the corpora lutea number with a concomitant increase in follicular cysts (Figure 6). The fourth generation presented a partial reversion in the ovarian follicular development.

## 4. Discussion

We found that the exposure of pregnant rats to sympathetic stress affects the reproductive function of the following four generations. This is probably due to the previously described chronic increase in NE due to stress, which affects the placental NE transport, leading to a decreased capacity of the placenta to clear NE from the circulation of the fetus to the mother [13]. This cold stress paradigm is a sympathetic stress in which there is no compromise of the ACTH axis [6,8,27]; thus, the effects found in this work could be ascribed to the overexposure of the fetus to increased NE levels that produce modifications in the processes affecting follicular development and ovulation. We previously used the chronic exposure to cold as an experimental tool to chronically stimulate sympathetic nerves in the ovaries of adult rats either by 3, 4, or 8 weeks [14,28,29]. In addition, we also used this stress paradigm in pregnant rats to study the impact on the ovary function of the progeny [16]. Now, the main objective of this paper was to know if cold stress modified the reproductive capacity of the following generations.

The estrus cycle in mammals is the reflex of a combination of events over time affecting the hypothalamus, pituitary, and the ovary [30,31]. A great amount of evidence suggests that sympathetic nerves arriving to the ovary also participate, controlling ovarian steroid secretion and follicular development and acting in a complementary manner to the ovulatory process. In this case, to explain the changes in estrus cycle activities, we have to consider both components. In either case, it is clear that during the night of proestrus in conjunction with the ovulation, copulation occurs in the rat [32]. At the same time, a hormone-dependent mechanism occurs [33,34].The fact that no changes were found in these behavioral events seems to suggest that the first and second generations of rats did not have profound changes in the hormonal control of the ovulatory event. It is interesting, however, to see that the fertile capacity was modified during the second generation of rats. This result could be explained by decreased ovulatory events independent of the proestrus stage (i.e., an ovulatory event). They might be the results of a poor control of the development of preovulatory follicles expressed as the decrease in the percentage of rats that were not pregnant even when they received the male (see Figure 3, second generation). This effect was more evident in the third generation, when only three out of nine rats accepted the male; another one accepted the male, but it did not get pregnant, and the others did not accept the male. This implies the existence of the possibility that autonomic sympathetic nerve control was activated by cold stress. This activation is originated at the magnocellular neurons of the paraventricular area of the hypothalamus [28,35] and could affect neurons involved in the control of sexual behavior [36] and can also be affected through the different generations. The impairment of female sexual behavior, specifically proceptivity/sexual receptivity, has been associated with the action of progesterone in the hypothalamus of female rats [33,34,37]. It is noteworthy that stress may impair oocyte quality in women, including growth and maturation, and may even cause the aging of oocytes [38]. None of these studies have been conducted in rats or in other species. It is interesting to note that the fourth generation, although recovering the capacity to be pregnant, did not recover the number of newborn rats. It seems that there could be two different mechanisms at play: one affecting the number of follicles able to ovulate (probably depending on the sympathetic activity) and the other related to the whole procedure of receptivity in which there is a participation of the reproductive hypothalamus. 

The local effect of stress at the ovary levels affected the follicular development over the generations as seen by the morphological analysis, and this is suggested in the uterus modification of the new follicular pool of at least two progenies of fetal ovaries. The fact that the number of healthy antral follicles declined without modifying follicular atresia directly affected the rate of ovulation as suggested by the continuous decrease in the number of corpus luteum. In addition, the total number of follicles (healthy, atretic, corpus luteum, and cyst) remained similar over the different generations, with the relative distribution between the generations having the clearest effect, going from a healthy reproductive process to a non-ovulatory process favoring the development of ovaries with a polycystic phenotype. Most probably, the mechanisms regulating the progression from preantral to ovulatory follicles were affected, and they were at least maintained during the four generations. Probably, epigenetic mechanisms are involved in these changes, such as the ones previously described as occurring after prenatal exposure to steroids [39]. Recently, a paper was published on ovary function and cold stress [40]. The authors used swimming in cold water, and they found an initial decrease in steroids hormones and a tendency to recover at longer times, probably by a compensatory effect of corticosterone [41]. It was also associated with changes in estrous cycle and ovary circulation. Unfortunately, there are no studies on the impact on next generations.

Overall, this is the first attempt to follow a long-term study of reproductive capacity over four generations and to describe the consequences of the modification in the uterus on the reproductive capacity of the progenies. One study of three generations of humans conducted by Sir-Petermann et al. studied the effect of a hyperandrogenic state during the gestation of mothers with PCOS [42,43,44,45]. They found many changes in the progeny that also affected the reproductive capacity both in the male and female progeny. Current evidence suggests that androgens could be one of the factors involved in the transgenerational effect of PCOS [46,47]. In addition, we found that in women with PCOS and in animal models with PCO [10,13], there is a hyper noradrenergic state that changes the placental transport of NE and thus exposes the fetus to increased levels of NE. The most important observation is that NE is the only neurotransmitter able to directly stimulate the androgen secretion from the ovary [12], and thus, it could be a common origin for the deranged reproductive function. Now, we confirm that NE exposure affects the reproductive capacity of the progeny, and opening studies to understand the mechanism of follicular development, especially in the earlier stage of development, when the process is independent of hypothalamic reproductive hormones, and in studies with environmental contaminants of both chemical or sensory origin and their impact on transgenerational diseases. 

One of the most important observations of this study was the impact that gestational sympathetic stress has on the reduced capacity of the second and third generations of female rats to be fertile after mating with a fertile male. Another alternative explanation could be that during stress exposure, the F0 generation contains the developing F1 females, who in turn contain developing F2 eggs, meaning F0, F1, and F2 all have direct exposure to a stressful environment. Meanwhile, the F3 generation will not have direct exposure to stress. As such, the F4 generation is the first born to females with no direct exposure to stress during development [48]. In a previous study, we described how after the same gestational stress paradigm, the newborn females rats of the first generation presented delayed development of primary and secondary follicles and a lower response to FSH and β-adrenergic receptors agonist to stimulate secondary follicles’ development and hence a delayed puberty [16].

## 5. Conclusions

We described that the exposure of gestating rats to sympathetic stress affects the fertility of the female progenies of the following generations. Most probably, these effects are the results of simultaneous exposure to NE up to the F3 generation during the intrauterine development. Probably some of the effects in F4 generation could be the results of epigenetic modifications.

## Data Availability

The data presented in this study are available on request from the corresponding author. The data are not publicly available because they are part of a continuity study.
